# Biomimetic Dual-Strategy Adaptive Differential Evolution for Joint Kinematic-Residual Calibration with a Neuro-Physical Hybrid Jacobian

**DOI:** 10.3390/biomimetics11030217

**Published:** 2026-03-18

**Authors:** Xibin Ma, Yugang Zhao, Zhibin Li

**Affiliations:** 1 School of Intelligent Science and Technology, Hangzhou Institute for Advanced Study, University of Chinese Academy of Sciences, Hangzhou 310024, China; max.ma@ucas.ac.cn; 2 Hangzhou ECON Science and Technology Co., Ltd., Hangzhou 310015, China; yugangzhao@econ-group.com; 3 School of Software Engineering, Chengdu University of Information Technology, Chengdu 610225, China; 4 Dazhou Key Laboratory of Government Data Security, Sichuan University of Arts and Science, Dazhou 635000, China

**Keywords:** biomimetic optimization, brain-body co-adaptation, sensorimotor control, Dual-Strategy Adaptive Differential Evolution, co-evolutionary calibration, Hybrid Jacobian

## Abstract

Improving absolute accuracy in industrial manipulators remains difficult because rigid-body kinematic calibration cannot fully represent configuration-dependent non-geometric effects. Drawing inspiration from biological brain–body co-adaptation, this study presents an Evolutionary Neuro-Physical Hybrid (Evo-NPH) framework in which rigid geometric parameters and neural compensator weights are treated as a single co-evolving decision vector. In the offline phase, a Dual-Strategy Adaptive Differential Evolution (DS-ADE) optimizer performs global joint identification using complementary exploration–exploitation behaviors and success-history inheritance, analogous to morphology-control co-evolution in biological systems. In the online phase, a Neuro-Physical Hybrid Jacobian (NPHJ) solver augments the analytical Jacobian with gradients from a Graph Kolmogorov–Arnold Network (GKAN), enabling sensorimotor-like real-time compensation on the learned physical manifold. Experiments on an ABB IRB 120 manipulator with 600 configurations (500 training, 100 testing) report a testing distance-residual RMSE of 0.62 mm, STD of 0.59 mm, and MAX of 0.83 mm. Relative to the uncalibrated baseline, RMSE is reduced by 86.75%; compared with the strongest published baseline, RMSE improves by 23.46%. Ablation results show that joint DS-ADE optimization outperforms a sequential pipeline by 32.6%, and the graph-structured KAN outperforms a parameter-matched MLP by 26.2%. Wilcoxon signed-rank tests (p<0.001) confirm statistical significance.

## 1. Introduction

As the lynchpin of the modern industrial paradigm, robotic systems have become inextricably integrated into the fabric of intelligent manufacturing, facilitating complex automated workflows ranging from logistics to precision medical interventions [1,2]. Despite their operational versatility and high repeatability, the absolute volumetric positioning accuracy [3] of serial manipulators remains a persistent bottleneck, often lagging orders of magnitude behind their resolution. This discrepancy is primarily attributed to a confluence of geometric errors arising from manufacturing tolerances and assembly misalignments, as well as complex non-geometric factors such as structural elasticity, gear backlash, and thermal deformation [4]. In high-precision scenarios, these errors accumulate along the kinematic chain, severely compromising the fidelity of the end-effector pose and necessitating rigorous calibration strategies [5].

To mitigate these deviations, robot calibration [6] has emerged as an imperative procedure. Conventional approaches typically rely on high-precision metrology equipment, such as laser trackers [7], ball bars [8], and electronic theodolites [9]. While these instruments offer superior measurement fidelity, their prohibitive cost and complex setup requirements render them inaccessible for many small-to-medium enterprises (SMEs). Consequently, there is a growing demand for algorithmic compensation strategies that can leverage cost-effective sensors, such as draw-wire encoders [10], to achieve high-accuracy calibration without substantial hardware investments.

In recent years, data-driven methodologies have demonstrated remarkable potential in modeling non-geometric errors. Standard approaches utilized in previous studies [11,12,13,14,15,16,17], including Back Propagation Neural Networks (BPNN) and Radial Basis Function Networks (RBFNN), map joint configurations to error residuals and act as black-box compensators. Despite their empirical success, these conventional architectures face two fundamental limitations. First, they exhibit topological agnosticism, meaning that standard Multi-Layer Perceptrons (MLPs) treat the manipulator’s joint state as a flat and unstructured vector. This approach ignores the inherent serial chain isomorphism where proximal link perturbations cascade to distal effectors [18]. Second, MLPs suffer from spectral bias, often struggling to capture high-frequency physical deformations without excessive parameterization [19]. Recently, Kolmogorov–Arnold Networks (KANs) have been proposed as a promising alternative utilizing learnable spline-based activation functions on edges to offer superior approximation capabilities and interpretability [20]. However, the integration of KANs with the topological structure of robotic kinematics remains an unexplored frontier.

In nature, precise motor behavior is achieved not by perfect rigid morphology alone, but by seamless co-adaptation of skeletal structure and neural control. Inspired by this biological paradigm, we argue that robotic calibration should avoid separating geometric identification from residual learning. Instead, morphology-like geometric parameters and control-like neural compensators should be optimized in a coupled manner so that structural representation and error compensation evolve consistently.

Furthermore, a critical methodological gap exists in the integration of learned error models with Inverse Kinematics (IK) solvers. Existing strategies predominantly function as post-process correctors where the predicted error is simply subtracted from the target pose. Crucially, the underlying IK solver, typically based on the Levenberg–Marquardt (LM) algorithm [21], continues to rely on the rigid-body Jacobian derived from nominal kinematics. In workspace regions exhibiting significant compliance or non-linear deformation, this rigid Jacobian fails to represent the true Fréchet derivative of the physical system, leading to suboptimal descent directions and potential convergence instability. There is, therefore, an urgent need for a differentiable solver that can synergize analytical kinematic constraints with learned neural gradients to guide the optimization process.

To overcome the aforementioned limitations, this paper presents a two-phase calibration framework termed Evo-NPH (Evolutionary Neuro-Physical Hybrid), with DS-ADE as the core bio-inspired optimization engine. In the first phase, a Dual-Strategy Adaptive Differential Evolution (DS-ADE) algorithm jointly identifies rigid-body D-H parameters and Graph Kolmogorov–Arnold Network (GKAN) weights in a unified search space, reducing the local-minimum sensitivity of sequential identification. In the second phase, a Neuro-Physical Hybrid Jacobian (NPHJ) solver leverages frozen optimized parameters for high-precision inverse kinematics control by combining analytical Jacobian terms with neural gradients from the trained GKAN. The principal contributions are as follows:

- We propose a bio-inspired DS-ADE engine for joint calibration, where geometric D-H parameters and neural compensator weights are optimized as a unified decision vector. The dual-strategy mutation and success-history adaptation improve exploration–exploitation balance and reduce local-minimum sensitivity in high-dimensional calibration landscapes.
- We formulate a Hybrid Jacobian solver for real-time inverse kinematics that, within each damped least-squares iteration, fuses the gradient field of the trained GKAN with the analytical kinematic Jacobian. The neural gradient term adaptively corrects the descent direction to capture compliance- and thermally induced residuals beyond the rigid-body Jacobian. In the dedicated solver comparison, the hybrid formulation achieves the highest success rate (98%), the fewest average iterations (5.7), and the lowest final residual (0.58 mm), demonstrating the benefit of combining rigid-body structure with learned neural compensation during online correction.
- We realize a continuous error manifold using compactly supported spline activations, making the learned compensation surface smooth to arbitrarily high order. Unlike the global weight couplings in standard perceptron layers, the local spline parameterization bounds each basis function’s influence radius, mitigating overfitting to measurement noise and preserving the gradient regularity required by joint optimization. Across 100 unseen configurations, the method attains a test RMSE of 0.62 mm, an STD of 0.59 mm, and a maximum error of 0.83 mm; Wilcoxon signed-rank tests confirm significance over all seven baselines (p<0.001).

## 2. Rigid-Body Kinematic Modeling and Geometric Parameter Identification

### 2.1. Analytical Modeling via the D-H Convention

**Figure 1:**
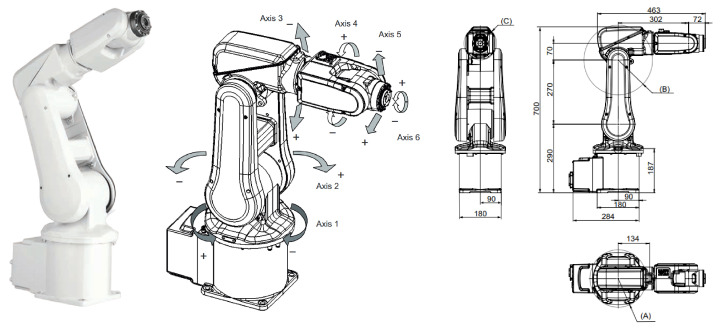
Kinematic topology and coordinate frame assignment of the experimental ABB IRB 120 manipulator. Labels A–C denote the top-view callout (A), enlarged side detail (B), and front-view callout (C) in the manufacturer’s drawing.

To construct the rigid-body backbone of the proposed Neuro-Physical framework, we employ the classical Denavit–Hartenberg (D-H) convention [22,23]. This method utilizes homogeneous transformation matrices to rigorously formalize the geometric topology of the serial manipulator. The six-axis ABB IRB 120 industrial robot utilized in this study is depicted in Figure 1, and its nominal kinematic parameters are detailed in Table 1. Unlike conventional calibration approaches that attempt to absorb all system uncertainties into geometric parameters, our framework strictly enforces a separation of concerns: the D-H model is dedicated solely to identifying static geometric deviations (e.g., link lengths and zeros), while complex non-geometric effects such as structural compliance and transmission errors are treated as residual dynamics to be explicitly captured by the subsequent Graph Kolmogorov–Arnold Network (GKAN).

Based on this rigid formulation, the forward kinematic chain is established. The homogeneous transformation matrix Tii−1, describing the spatial relationship between link *i* and i−1, is mathematically expressed as

(1) Tii−1=cosθi−sinθicosαisinθisinαiaicosθisinθicosθicosαi−cosθisinαiaisinθi0sinαicosαidi0001

where $a_{i}$ denotes the link length, $d_{i}$ represents the link offset, $\alpha_{i}$ specifies the twist angle, and $\theta_{i}$ is the joint variable. In the parameter identification phase, we identify a static zero-offset correction $\Delta \theta_{0,i}$ for each joint. The nominal zero-offset $\theta_{i}^{\mathrm{nom}}$ (from Table 1) is updated as

(2) $$\theta_{0,i}^{\mathrm{cal}}=\theta_{i}^{\mathrm{nom}}+\Delta \theta_{0,i},$$

Consequently, the actual joint angle utilized in the computation is

(3) $$\theta_{i}=q_{k,i}+\theta_{0,i}^{\mathrm{cal}},$$

where $q_{k,i}$ is the encoder reading.

By successively multiplying these transformation matrices along the serial chain, the global pose of the end-effector with respect to the base frame is derived as

(4) Ttotal=∏i=16Tii−1,

The Cartesian coordinates of the end-effector $P_{\mathrm{end}}$ are extracted from the translational component of the final matrix:

(5) $$P_{\mathrm{end}}\left(\Psi ,q_{k}\right)={\left[{\left(T_{total}\right)}_{1,4},\;{\left(T_{total}\right)}_{2,4},\;{\left(T_{total}\right)}_{3,4}\right]}^{T}\in R^{3}.$$

The observation model is defined by the Euclidean distance between this predicted position and the fixed encoder base point $P_{0}$:

(6) $$h\left(\Psi ,q_{k}\right)={∥P_{\mathrm{end}}\left(\Psi ,q_{k}\right)-P_{0}∥}_{2}.$$

Accordingly, the kinematic residual $\varepsilon_{k}$ at the *k*-th configuration is formulated as the discrepancy between the measured cable length $z_{k}$ and the model prediction:

(7) $$\varepsilon_{k}=z_{k}-h\left(\Psi ,q_{k}\right).$$

Here, $\Psi \in R^{24}$ represents the vector of geometric parameters to be identified, constructed by stacking the deviations for all joints: $\Psi ={\left[\Delta a_{1},\Delta d_{1},\Delta \alpha_{1},\Delta \theta_{0,1},\ldots ,\Delta \theta_{0,6}\right]}^{T}$. The calibrated parameters are obtained by updating the nominal values with these deviations. It is crucial to note that the residual term $\varepsilon_{k}$ contains deterministic non-geometric errors (e.g., structural compliance) that the rigid-body D-H model inherently fails to resolve. In our proposed framework, rather than treating these residuals as noise or fitting them in a separate post-processing stage, we integrate them into a unified objective function, where the rigid geometric parameters and the Graph Kolmogorov–Arnold Network (GKAN) are jointly optimized to minimize the total kinematic error.

### 2.2. Global Joint Optimization via Dual-Strategy Adaptive Differential Evolution

Conventional gradient-based calibration methods typically rely on a sequential optimization paradigm, where geometric parameters are identified first, followed by residual network training. This decoupled approach is prone to error accumulation and often converges to local minima due to the highly non-convex landscape of the kinematic error surface. To address these limitations, we adopt Differential Evolution (DE)-style global search [24] and integrate adaptive success-history mechanisms inspired by JADE/SHADE [25,26] and L-SHADE [27] into a Dual-Strategy Adaptive Differential Evolution (DS-ADE) algorithm. This offline calibration engine simultaneously optimizes rigid-body geometric parameters and neural network weights in a unified search space.

#### 2.2.1. Unified Decision Vector and Objective Function

The calibration problem is cast as a global optimization task over a high-dimensional decision vector Θ. This formulation enables the solver to co-evolve the physical and neural representations of the robot. From an embodied-intelligence perspective, the geometric D-H parameters Ψ represent skeletal morphology, while the network weights $W_{GKAN}$ represent a neuromuscular-style compensator. Concatenating these terms into a unified variable establishes a mathematical analog of brain–body co-evolution, ensuring that neural compensation is learned in direct correspondence with the identified physical structure. The vector is constructed by concatenating the rigid-body D-H parameters Ψ and the flattened spline coefficients $W_{GKAN}$ of the neural network:

(8) $$\Theta ={\left[\Psi^{⊤},\;\mathrm{vec}{\left(W_{GKAN}\right)}^{⊤}\right]}^{⊤}\in R^{D},$$

where *D* denotes the total dimensionality of the search space. For the ABB IRB 120 case studied here, the resulting unified decision vector has dimension greater than 200, since the 24 geometric parameters are optimized jointly with the flattened spline coefficients of the compact GKAN. This high-dimensional setting is one reason for preferring archive-assisted adaptive DE over a purely local deterministic search scheme.

The fitness function J(Θ) is designed to minimize the kinematic residual while strictly enforcing physical plausibility through regularization. The objective functional synthesizes four distinct components:

(9) $$J\left(\Theta \right)=L_{RMSE}\left(D\right)+\lambda_{max}L_{Max}\left(D\right)+\lambda_{DH}∥W_{DH}^{-1}{\Psi ∥}_{2}^{2}+\lambda_{Res}{∥e_{nn}∥}_{2}^{2}.$$

Here, given a dataset $D={\left\{\left(q_{k},z_{k}^{meas}\right)\right\}}_{k=1}^{N}$ obtained from the draw-wire sensor, the error metrics are defined based on the scalar distance residuals:

(10) $$\begin{array}{cc} L_{RMSE} & =\sqrt{\frac{1}{N}\sum_{k=1}^{N}{\left(z_{k}^{meas}-{∥P_{est}\left(q_{k};\Theta \right)-P_{0}∥}_{2}\right)}^{2}}, \end{array}$$

(11) $$\begin{array}{cc} L_{Max} & =\max_{k}\left|z_{k}^{meas}-{∥P_{est}\left(q_{k};\Theta \right)-P_{0}∥}_{2}\right|. \end{array}$$

The term $\lambda_{DH}$ penalizes excessive deviations in geometric parameters to prevent physical implausibility, while $\lambda_{Res}$ constrains the magnitude of the neural compensation to avoid overfitting high-frequency noise. The matrix $W_{DH}$ represents the diagonal scaling factors for the D-H parameters. In the present implementation, the selected regularization pair $\left(\lambda_{DH},\lambda_{Res}\right)=\left(10^{-4},5\times 10^{-2}\right)$ imposes a conservative bias toward physically plausible geometric corrections while preserving sufficient flexibility for the neural residual branch.

#### 2.2.2. Biomimetic Rationale: Dual Behaviour and Success-History Inheritance

From a biomimetics perspective, each candidate solution is treated as an individual genotype, while the whole population adapts through variation and selection. The two mutation strategies represent complementary foraging behaviors: an exploitative mode (via current-to-pbest/1) that intensifies search around high-fitness elites, and an exploratory mode (via rand-to-pbest/1) that preserves diversity and expands search coverage. The success-history memory used to update (F,CR) and strategy probability can be interpreted as inherited behavioral tendency, where traits that repeatedly improve fitness are preferentially expressed in subsequent generations. This mechanism improves robustness against stagnation in rugged, multimodal calibration landscapes. It is particularly important for the coupled geometry-network search space, where physical structure and neural compensation must adapt jointly without falling into evolutionary dead-ends.

#### 2.2.3. Dual-Strategy Search and Adaptation Mechanism

To effectively navigate the high-dimensional landscape (typically D>200) and mitigate the risk of stagnation, the DS-ADE algorithm employs a multi-operator mechanism. Two distinct mutation strategies are integrated to balance local exploitation and global exploration. For the *i*-th target vector $x_{i,g}$ at generation *g*, a mutation strategy is selected probabilistically.

To accelerate convergence, the algorithm primarily utilizes the current-to-pbest/1 strategy, which guides the search toward the superior sub-population. The mutant vector $v_{i,g}^{\left(1\right)}$ is generated as

(12) $$v_{i,g}^{\left(1\right)}=x_{i,g}+F_{i}\cdot \left(x_{pbest,g}-x_{i,g}\right)+F_{i}\cdot \left(x_{r1,g}-{\tilde{x}}_{r2,g}\right).$$

Conversely, to enhance global exploration and escape local optima, the rand-to-pbest/1 strategy is employed, which introduces diversity by utilizing a random base vector:

(13) $$v_{i,g}^{\left(2\right)}=x_{r0,g}+F_{i}\cdot \left(x_{pbest,g}-x_{r0,g}\right)+F_{i}\cdot \left(x_{r1,g}-{\tilde{x}}_{r2,g}\right).$$

In these formulations, $x_{pbest,g}$ is randomly chosen from the top p% superior individuals, $x_{r0,g}$ and $x_{r1,g}$ are selected from the current population $P_{g}$, and ${\tilde{x}}_{r2,g}$ is selected from the union of the population and an external archive $P_{g}\cup A_{g}$. The external archive $A_{g}$ acts as a genetic memory (supplementary gene pool) that preserves diversity and supports long-range variation. The indices satisfy the condition r1≠r2≠i.

The probability of selecting the first strategy, denoted as $p_{1}$, is dynamically adapted based on the success history. If learning experiences indicate that one strategy yields larger fitness improvements, its selection probability is increased according to

(14) $$p_{1,g+1}=\frac{\sum_{k\in S_{1}}\Delta J_{k}}{\sum_{k\in S_{1}}\Delta J_{k}+\sum_{k\in S_{2}}\Delta J_{k}},$$

where $S_{1}$ and $S_{2}$ are the sets of individuals successfully advanced by the first and second strategies, respectively, and $\Delta J_{k}$ represents the fitness gain. Although no additional exponential smoother is imposed on $p_{1}$, abrupt oscillations are mitigated because the update aggregates the cumulative fitness gains of all successful individuals within a generation rather than reacting to a single offspring, and because the companion adaptation of (F,CR) is filtered through the success-history memory M across successive generations.

Concurrently, the control parameters *F* and CR undergo self-adaptation to align with the topological characteristics of the kinematic manifold. The scaling factor $F_{i}$ and crossover rate $CR_{i}$ for each individual are sampled independently from Cauchy and Normal distributions, respectively:

(15) $$F_{i}=\mathrm{Cauchy}\left(\mu_{F},0.1\right),\;CR_{i}=N\left(\mu_{CR},0.1\right).$$

The location parameters $\mu_{F}$ and $\mu_{CR}$ are updated using a memory M that records successful configurations. The memory update follows a weighted Lehmer mean:

(16) $$\mu_{F,new}=\frac{\sum_{k\in S}w_{k}\cdot F_{k}^{2}}{\sum_{k\in S}w_{k}\cdot F_{k}},\;w_{k}=\frac{\Delta J_{k}}{\sum_{j\in S}\Delta J_{j}}.$$

Following mutation and parameter generation, a binomial crossover is performed to generate the trial vector $u_{i,g}$. Selection is strictly greedy, where the trial vector replaces the target vector only if $J\left(u_{i,g}\right)\leq J\left(x_{i,g}\right)$.

#### 2.2.4. Operational Usage: Offline Calibration

In the practical workflow, this global optimization phase is executed offline using a collected dataset of joint configurations and corresponding end-effector measurements. The DS-ADE algorithm iterates through generations until the fitness function converges or a maximum iteration count is reached. The output is the optimal parameter set $\Theta^{*}$, which defines the calibrated geometric backbone and the frozen weights of the GKAN model, ready for deployment.

### 2.3. Real-Time Control via Neuro-Physical Hybrid Jacobian

While the DS-ADE algorithm provides a robust global solution for offline parameter identification, its stochastic nature and high computational cost make it unsuitable for online control loops, which typically require millisecond-level response times. To bridge the gap between accurate calibration and fast execution, we leverage the differentiability of the trained GKAN to construct a Neuro-Physical Hybrid Jacobian (NPHJ) solver. This solver functions as the online execution engine, enabling real-time inverse kinematics compensation.

#### 2.3.1. Hybrid Forward Kinematics

Once the optimal parameters $\Theta^{*}$ are identified and frozen, the forward kinematic model becomes a deterministic function. For a target trajectory point $p_{target}$, the inverse kinematics problem seeks the joint configuration q that minimizes the tracking error:

(17) $$\min_{q}L_{track}\left(q\right)=\frac{1}{2}{∥p_{target}-P_{est}\left(q;\Theta^{*}\right)∥}_{2}^{2}.$$

where $P_{est}$ is the superposition of the calibrated rigid D-H model and the learned GKAN non-geometric compensation.

#### 2.3.2. Derivation of the Hybrid Jacobian

To solve this optimization problem efficiently using gradient descent, the total differential of the estimated position with respect to the joint angles q is derived. The Hybrid Jacobian $J_{hybrid}\in R^{3\times 6}$ is defined as the sum of analytical and neural gradients:

(18) $$J_{hybrid}\left(q\right)=\frac{\partial K_{rigid}}{\partial q}+\frac{\partial N_{GKAN}}{\partial q}.$$

Biologically, precise motor behavior is generated by fusing rigid biomechanical constraints and adaptive neuromuscular adjustments. In this sense, $J_{hybrid}$ models a sensorimotor reflex: $\partial K_{rigid}/\partial q$ provides the hard skeletal constraint, while $\partial N_{GKAN}/\partial q$ provides anticipatory neural-style compensation for compliance and thermal effects.

The first term is the analytical Jacobian of the rigid skeleton, derived via the vector cross-product method. For the *j*-th revolute joint, the column vector is

(19) $$J_{phy,j}=z_{j-1}\times \left(p_{ee}-p_{j-1}\right).$$

The second term, the Neural Jacobian, is computed via automatic differentiation. Since the trained GKAN is a composition of differentiable spline operations, its gradient is obtained by backpropagating through the computational graph:

(20) $$J_{nn,j}=\sum_{k}\frac{\partial e_{nn}}{\partial h_{k}^{\left(L\right)}}\ldots \frac{\partial h_{1}^{\left(0\right)}}{\partial q_{j}}.$$

This term captures the sensitivity of the learned non-geometric error manifold to configuration changes, allowing the solver to anticipate and correct for structural compliance.

#### 2.3.3. Operational Usage: Online Compensation

For real-time control, the Levenberg-Marquardt algorithm is employed to iteratively update the joint angles. At control step *k*, the update rule is given by solving the damped normal equations:

(21) $$\left(J_{hybrid}^{⊤}J_{hybrid}+\lambda I\right)\Delta q_{k}=J_{hybrid}^{⊤}\left(p_{target}-P_{est}\left(q_{k}\right)\right).$$

The configuration is updated as

(22) $$q_{k+1}=q_{k}+\alpha \cdot \Delta q_{k}.$$

In all experiments, the damping factor is fixed at $\lambda =10^{-2}$ (Table 2). This choice provides stable regularization near ill-conditioned Jacobians without introducing noticeable conservatism in the local update; an explicitly adaptive damping schedule remains an important extension for future work. By utilizing the Hybrid Jacobian, the solver corrects the descent direction based on the true physical manifold learned by the network. In practice, this solver is embedded within the robot controller. It receives the target Cartesian coordinate $p_{target}$ and outputs the compensated joint angles $q_{cmd}$ to the servo drives, ensuring high-fidelity positioning accuracy with minimal computational latency.

### 2.4. Design and Analysis of the DS-ADE Driven Neuro-Physical Framework

**Figure 2:**
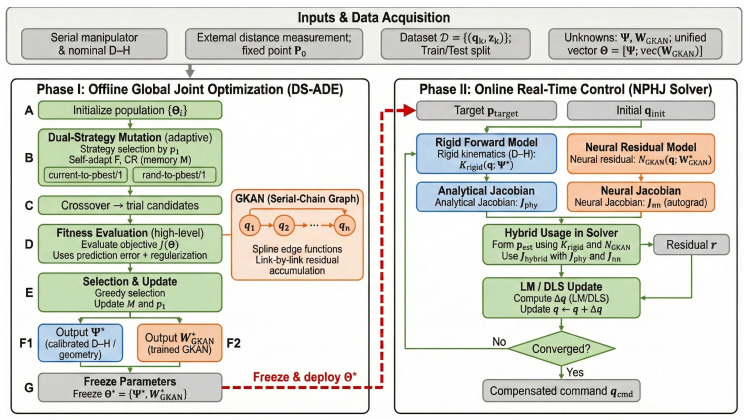
Overview of the proposed Evo-NPHframework. Phase I performs offline global joint optimization using DS-ADE (mathematically formulating brain-body co-adaptation), and Phase II executes online real-time compensation using the NPHJ solver, where analytical and neural Jacobians emulate a sensorimotor reflex. Labels A–E denote initialization, dual-strategy mutation, crossover, fitness evaluation, and selection/update, respectively; F1 and F2 indicate the calibrated geometric output and the trained GKAN output; G denotes freezing the optimized parameters before controller deployment. The red dashed arrow denotes the deployment of the frozen optimized parameters to the controller.

To provide a comprehensive visualization of the proposed calibration strategy, the complete workflow is illustrated in Figure 2. Unlike traditional multi-stage pipelines that propagate errors from geometric identification to residual learning, the proposed architecture is structured into two distinct operational phases: an offline global joint optimization phase driven by the DS-ADE algorithm, and an online real-time control phase empowered by the NPHJ solver.

The process initiates with Phase I: Offline Global Joint Optimization. In this phase, the system inputs include the nominal D-H parameters, the measurement dataset D, and the search bounds for the unified decision vector. Rather than isolating geometric and non-geometric errors, the DS-ADE algorithm treats the calibration as a holistic co-evolutionary process. A population of candidate vectors, each encoding both rigid-body parameters and neural spline coefficients, evolves through the dual-strategy mutation mechanism. The current-to-pbest/1 strategy accelerates convergence by exploiting gradient information inherent in the population, while the rand-to-pbest/1 strategy maintains diversity to explore the multimodal landscape. Through adaptive parameter adjustment and greedy selection, the algorithm iteratively refines the population until the global optimum $\Theta^{*}$ is identified. This step effectively establishes a unified kinematic model that optimally balances physical interpretability and data-driven compensation without the bias introduced by sequential fitting.

Upon convergence, the workflow transitions to Phase II: Online Real-Time Control. As visually highlighted in Figure 2, the optimized parameters $\Theta^{*}$ are frozen and transferred to the robot controller. The computational engine shifts from stochastic search to deterministic gradient-based solving. The NPHJ solver integrates the analytical Jacobian from the rigid backbone with the neural Jacobian derived via automatic differentiation of the GKAN. This hybrid gradient guides the Levenberg–Marquardt optimizer to compute the exact joint configuration for a given target pose. By leveraging the differentiability of the trained network, this phase ensures high-fidelity trajectory tracking with millisecond-level latency, bridging the gap between complex offline learning and fast online execution. The detailed algorithmic steps are provided in Table 3.

The computational complexity of the proposed framework is analyzed with respect to its two operational modes. For the offline optimization phase (Phase I), the computational cost is dominated by the population-based fitness evaluation. Let *N* denote the dataset size, NP the population size, and $G_{max}$ the maximum number of generations. The complexity scales as $O(G_{max}\cdot NP\cdot N\cdot C_{FK})$, where $C_{FK}$ represents the cost of one hybrid forward kinematic evaluation. Although this phase is computationally intensive, it is executed strictly offline, and the DS-ADE algorithm ensures that the solution quality is not compromised by local minima. For the online control phase (Phase II), the complexity is determined by the iterative Jacobian assembly and linear system solution. The calculation of the analytical Jacobian is O(n), while the neural Jacobian via backpropagation scales with the network depth and spline grid size as O(L·G). Solving the linear system for the 6-DOF manipulator takes constant time $O(n^{3})$. Thus, the total complexity for $T_{iter}$ control iterations is $O(T_{iter}\cdot \left(n+L\cdot G\right))$. Since n,L,G are small constants, this ensures that the complex hybrid model runs in real-time, independent of the large offline training dataset size.

## 3. Experiments and Results

### 3.1. Experimental Setup and Data Acquisition

To validate the efficacy of the proposed neuro-physical calibration strategy in a realistic industrial setting, an experimental platform was established using an ABB IRB 120 manipulator (ABB Ltd., Zurich, Switzerland). This six-degree-of-freedom serial robot serves as a representative model for precision manufacturing tasks, featuring a compact kinematic chain with a maximum reach of 580 mm and a rated payload of 3 kg. Its pose repeatability is specified at ±0.01 mm, providing a stable baseline for evaluating the residual compensation performance of the algorithm.

#### 3.1.1. Metrology System Configuration

Ground-truth distance measurements were acquired using a high-precision draw-wire displacement encoder (Model: HY150-2000), which acts as the primary external observation device. The metrological characteristics of this sensor are detailed in Table 4. The sensor body was rigidly mounted at a fixed reference coordinate $P_{0}$ within the robot’s base frame, while the extensible wire was coupled to the tool center point (TCP) via a magnetic fixture. This setup enables the continuous monitoring of the Euclidean distance $z_{k}={∥p_{tcp}-P_{0}∥}_{2}$ across the robot’s workspace. The sensor offers a linearity of 0.05% FS over a 2000 mm range, ensuring sufficient resolution to capture minute kinematic deviations.

#### 3.1.2. Data Synchronization and Preprocessing

A custom data acquisition (DAQ) system was developed within the National Instruments LabVIEW environment (National Instruments, Austin, TX, USA) to ensure strict temporal synchronization between the robot controller and the external sensor. The joint configuration vector $q_{k}\in R^{6}$ was retrieved directly from the robot controller via the Ethernet/IP protocol. Simultaneously, the digital output from the displacement sensor was recorded via a dedicated counter interface at 1 kHz. To mitigate the effects of high-frequency measurement noise and mechanical vibrations, a stationary averaging protocol was implemented: for each calibration pose, the robot was held static for 2 s while the sensor stream was sampled continuously. A stability gate was applied to the stationary interval, from which 50 samples were selected, and the recorded measurement $z_{k}$ was computed as the arithmetic mean of these samples.

#### 3.1.3. Sampling Strategy

**Figure 3:**
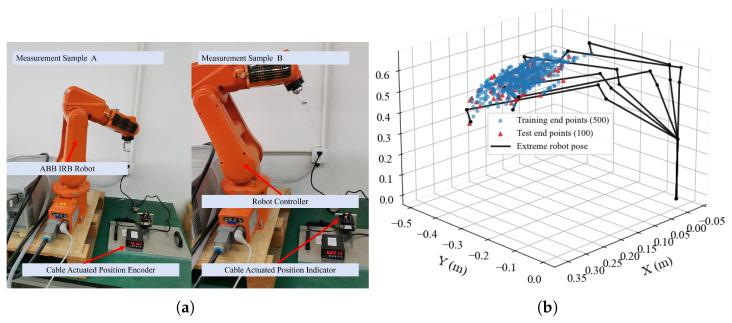
Robot workspace configuration and sampling distribution. (**a**) Schematic illustration of the draw-wire measurement setup in the robot workspace. (**b**) Spatial distribution of sampled configurations in the workspace (positions computed via nominal forward kinematics from recorded joint angles for visualization purposes), where blue circles denote training samples and red triangles denote testing samples.

To ensure the identified model possesses sufficient generalization capability, a total of N=600 distinct spatial configurations were collected. The sampling positions were generated using a deterministic quasi-random Halton sequence rather than a uniform grid. This approach avoids aliasing effects and ensures a more uniform coverage of the operational volume. For visualization only, the recorded joint configurations were projected into Cartesian space using nominal forward kinematics, yielding an apparent workspace coverage of approximately X∈[0,300] mm, Y∈[−500,−250] mm, and Z∈[350,550] mm relative to the base frame in Figure 3b. These ranges therefore describe nominally projected sampled configurations rather than externally measured 3D ground-truth positions. This diverse dataset allows the DS-ADE algorithm to effectively explore the global kinematic landscape during the offline optimization phase.

### 3.2. Implementation Details and Hyperparameter Settings

The numerical experiments were conducted within a Python 3.12 environment, utilizing the PyTorch 2.10.0 library for efficient differentiable tensor computations. All algorithmic procedures were executed on a workstation equipped with an Intel Core i5-13400F processor and 64 GB of RAM. To ensure a rigorous evaluation of the calibration performance, the acquired dataset consisting of 600 distinct spatial configurations was partitioned using a stratified random sampling strategy. Specifically, 500 samples (approximately 83.3%) were allocated to the training set for the global joint optimization of the unified parameter vector, while the remaining 100 samples (approximately 16.7%) were reserved as an independent testing set to verify the generalization capability of the model on unseen configurations.

The hyperparameters for the proposed framework were configured based on the specific kinematic characteristics of the IRB 120 manipulator. For the Graph Kolmogorov–Arnold Network (GKAN), a compact topology was adopted to mitigate the risk of overfitting, characterized by a depth of L=1 and a spline grid resolution of G=5 knots. This configuration enables the network to effectively capture low-frequency structural compliance errors while suppressing high-frequency measurement noise. For the Dual-Strategy Adaptive Differential Evolution (DS-ADE) algorithm, the population size was initialized at NP=48 to maintain sufficient diversity within the high-dimensional search space, with the optimization process terminating after $G_{max}=300$ generations. To reflect standard manufacturing tolerances, the search boundaries for the geometric parameters were constrained to ±10 mm for length offsets and ±5° for angular deviations. Furthermore, the regularization coefficients were set to $\lambda_{DH}=10^{-4}$ and $\lambda_{Res}=5\times 10^{-2}$ to enforce physical plausibility and penalize solutions that deviate excessively from the nominal kinematic model.

### 3.3. Performance Evaluation Metrics

To quantitatively assess calibration quality under draw-wire sensing, we report three statistics of the distance residual: Root Mean Square Error (RMSE), Standard Deviation (STD), and Maximum Absolute Error (MAX). RMSE is the primary global indicator of range-model mismatch, STD measures residual dispersion and stability, and MAX captures worst-case distance deviation for safety margin analysis.

Let $z_{k}$ denote the measured cable length and ${\hat{z}}_{k}$ denote the length predicted by the calibrated hybrid model for the *k*-th sample. Defining the residual error as $e_{k}=z_{k}-{\hat{z}}_{k}$, the evaluation metrics are formulated as follows:

(23) $$\mathrm{RMSE}=\sqrt{\frac{1}{n}\sum_{k=1}^{n}e_{k}^{2}},$$

(24) $$\mathrm{STD}=\sqrt{\frac{1}{n-1}\sum_{k=1}^{n}{\left(e_{k}-\bar{e}\right)}^{2}},$$

(25) $$\mathrm{MAX}=\max_{k=1,\;\ldots ,\;n}\left|e_{k}\right|,$$

where *n* represents the number of samples in the evaluation subset, and $\bar{e}$ denotes the arithmetic mean of the residuals.

### 3.4. Comparative Methods

To rigorously benchmark the performance of the proposed Evolutionary Neuro-Physical Hybrid (Evo-NPH) framework, seven distinct calibration strategies were selected from the literature. These baselines represent a broad spectrum of existing solutions, ranging from classical recursive estimators to advanced hybrid evolutionary algorithms and neural compensators. The summary of these comparative methods, including their core architectures and the specific rationale for their inclusion, is presented in Table 5.

The selected comparative strategies can be categorized into three primary classes based on their mathematical foundations. First, to quantify the baseline accuracy achievable by rigid-body kinematics alone, the industry-standard SGC-LM (M1) method [21] is included as the geometric reference. Second, to evaluate the efficacy of data-driven residual modeling, we employ ES-RBFNN (M2) [14] and KC-JVP (M7) [28], which utilize radial basis functions and deep feedforward networks, respectively, to map non-geometric errors and dynamic trajectory deviations. Third, to assess the convergence capability of global optimization strategies, a series of hybrid evolutionary and recursive estimators are selected. This group includes the recursive Bayesian EKF-PF (M3) [29], the two-stage sequential LM-PF (M4) [30], and the bio-inspired metaheuristic approaches EKF-DQBPSO (M5) [31] and ANN-BFPA (M6) [32]. Comparison against these state-of-the-art techniques serves to validate the core components of the Evo-NPH framework: specifically, the global search capability of the Dual-Strategy Adaptive Differential Evolution (DS-ADE) algorithm in offline identification, and the compensation precision of the Neuro-Physical Hybrid Jacobian (NPHJ) solver in real-time control.

**Table 5:** Summary of comparative calibration methods and benchmarking rationale.

ID	Method [Ref.]	Core Architecture	Benchmarking Rationale
M1	**SGC-LM** [21]	Standard Rigid-Body D-H Model identified via Levenberg–Marquardt (LM).	Serves as the industry-standard geometric baseline to quantify accuracy limits without non-geometric compensation.
M2	**ES-RBFNN** [14]	Error Similarity (ES) index combined with Radial Basis Function Neural Network.	Evaluates the efficacy of shallow, data-driven neural networks in mapping residuals compared to the proposed graph architecture.
M3	**EKF-PF** [29]	Hybrid estimator fusing Extended Kalman Filter (EKF) with Particle Filter (PF).	Benchmarks recursive Bayesian estimation techniques and their ability to mitigate linearization errors.
M4	**LM-PF** [30]	Two-stage strategy: LM for local convergence followed by PF for global refinement.	Assesses the performance difference between sequential hybrid strategies and the proposed simultaneous joint optimization.
M5	**EKF-DQBPSO** [31]	Integration of EKF with Dual Quantum-Behaved Particle Swarm Optimization.	Represents state-of-the-art metaheuristics; provides a baseline for evaluating convergence speed and global search capability.
M6	**ANN-BFPA** [32]	ANN trained via hybrid Butterfly and Flower Pollination Algorithm (BFPA).	Compares the effectiveness of different bio-inspired metaheuristics in optimizing neural compensators.
M7	**KC-JVP** [28]	Kinematic Calibration combined with Joint Variable Prediction (RPSO-DCFNN).	Evaluates the system’s ability to handle trajectory-dependent errors and dynamic variations under varying load conditions.

### 3.5. Experimental Results and Validation

Calibration performance of the proposed Evo-NPH framework was evaluated through experiments on an ABB IRB 120 six-axis industrial manipulator. Seven published algorithms covering geometric identification, recursive estimation, metaheuristic search, and data-driven compensation were selected as baselines: SGC-LM [21], ES-RBFNN [14], EKF-PF [29], LM-PF [30], EKF-DQBPSO [31], ANN-BFPA [32], and KC-JVP [28]. Their full names and selection rationale are provided in Table 5.

#### 3.5.1. Accuracy Comparison

**Figure 4:**
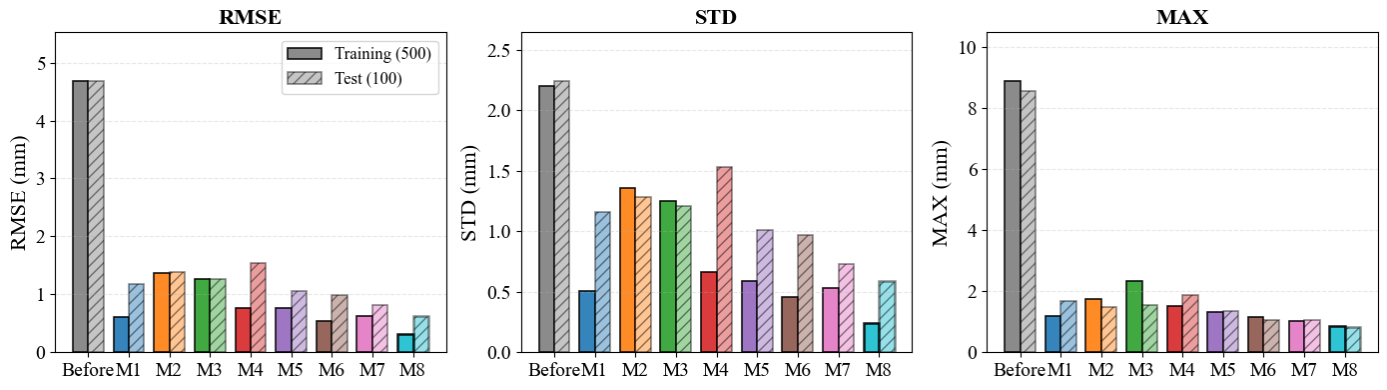
Comparative analysis of error metrics (RMSE, STD, and MAX) across the training (N=500) and testing (N=100) datasets. The solid bars represent the results on the training set, while the hatched semi-transparent bars denote the performance on the test set. The proposed Evo-NPH framework (M8) consistently exhibits the lowest error magnitudes across all three indicators, validating its superior generalization capability compared to baselines M1–M7.

The RMSE, STD, and MAX metrics for every method are listed in Table 6; the corresponding comparative bar charts for both training and testing datasets are visualized in Figure 4. Prior to calibration, the manipulator shows a test RMSE of 4.68 mm with a peak error reaching 8.55 mm—well beyond the tolerance envelope required for precision assembly. Every calibration algorithm examined here brings this error down substantially, which underlines the practical importance of kinematic parameter identification.

The geometric-only method SGC-LM (M1) lowers the test RMSE to 1.17 mm by identifying rigid-body D-H parameters, yet without a non-geometric compensation channel the residual error plateaus. Its training RMSE of 0.61 mm is roughly half the test value, pointing to limited extrapolation beyond the training workspace. ES-RBFNN (M2) produces a test RMSE of 1.38 mm; the purely data-driven architecture, lacking embedded kinematic structure, provides little advantage over model-based approaches when evaluated on unseen configurations. Within the recursive estimation category, EKF-PF (M3) reaches a test RMSE of 1.26 mm. Incorporating a particle filter alongside the EKF partially mitigates the first-order linearization bias, although the improvement over single-stage filters remains modest. The two-stage LM-PF (M4) exhibits the widest training–test discrepancy among all candidates: a training RMSE of 0.76 mm inflates to 1.54 mm on the test set, roughly doubling. This degradation arises because the LM stage is sensitive to its starting point; the resulting suboptimal parameters then propagate into the particle filter and narrow its effective search region. Metaheuristic-augmented methods deliver more competitive results. EKF-DQBPSO (M5) records a test RMSE of 1.06 mm by coupling state estimation with a dual quantum-behaved swarm search, at the expense of increased computational cost from maintaining two parallel populations. ANN-BFPA (M6) pushes the error further down to 0.99 mm through pollination-inspired weight optimization. KC-JVP (M7) ranks first among all baselines at a test RMSE of 0.81 mm, an STD of 0.73 mm, and a MAX of 1.06 mm, confirming the merit of joint-variable-level residual prediction. That said, the method is tightly coupled to specific trajectory data and offers no recursive state-space formulation for online deployment. An important observation from Table 6 is the discrepancy between training and test accuracy across methods. Besides the LM-PF case noted above, SGC-LM also nearly doubles from 0.61 mm to 1.17 mm. Such gaps suggest that a low training error does not, by itself, guarantee reliable calibration; robustness on unseen poses deserves equal attention.

The proposed Evo-NPH (M8) obtains the smallest error on every metric: a test RMSE of 0.62 mm, an STD of 0.59 mm, and a maximum error of 0.83 mm. Measured against the uncalibrated baseline, this constitutes an RMSE reduction of 86.75%. Relative to the top-performing baseline KC-JVP (0.81 mm), the improvement is 23.46%; relative to the geometric baseline SGC-LM (1.17 mm), it reaches 47.01%. The two-phase design underlies these gains: the DS-ADE stage supplies a globally informed initial parameter set via adaptive differential evolution, after which the NPHJ stage refines the estimate through a neurally parameterized Hybrid Jacobian that simultaneously accounts for geometric and non-geometric error sources in a single optimization pass.

**Figure 5:**
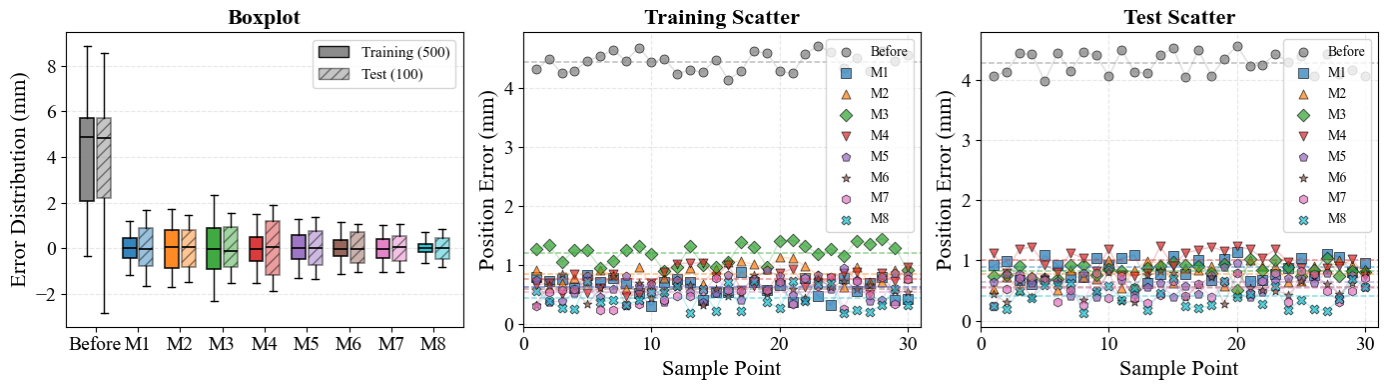
Statistical error distribution and sample-wise performance verification. The left panel presents box-and-whisker plots comparing the error distributions of the uncalibrated baseline and methods M1–M8 on both training and testing datasets, illustrating the dispersion and median accuracy. The middle and right panels visualize residual distance errors for a subset of sampled points from the training and testing sets, respectively, highlighting the stability of the proposed Evo-NPH framework (M8) compared to the fluctuations observed in stochastic baselines. In the left panel, the solid boxplots denote the training set and the hatched semi-transparent boxplots denote the test set; in the middle and right panels, the dashed horizontal lines indicate the mean residual level of each method over the displayed subset.

As illustrated in Figure 5, the proposed Evo-NPH framework exhibits superior statistical stability and consistency. The box-and-whisker plots (left panel) reveal that our method (M8) produces the most concentrated error distribution with the narrowest interquartile range and minimal outliers compared to stochastic baselines M6 and M7. Furthermore, the sample-wise scatter plots for both training (middle panel) and testing (right panel) datasets demonstrate that the residual errors of the proposed method fluctuate strictly within a compact band around zero, avoiding the large deviations observed in other hybrid approaches. This confirms that the joint optimization strategy, combined with the Neuro-Physical Hybrid Jacobian compensation, significantly enhances both calibration accuracy and reliability across the entire workspace.

#### 3.5.2. Computational Efficiency

Table 7 lists the iteration count and wall-clock time of each method, recorded on the full 600-sample dataset (500 training + 100 test) with a single-threaded implementation on an Intel Core i5-13400F CPU. Lightweight model-based approaches such as EKF-DQBPSO (M5) and ANN-BFPA (M6) terminate in 21.2 s and 23.0 s, respectively, because their per-iteration cost is dominated by a single forward–backward pass through a compact parameter space. The particle-based EKF-PF (M3) is the slowest at 161.0 s, reflecting the poor scalability of sequential Monte Carlo sampling in high-dimensional settings.

The proposed Evo-NPH (M8) requires 86 iterations and 61.11 s to converge—roughly 2.2× the runtime of the next-best baseline KC-JVP (M7, 27.5 s). This additional cost stems primarily from the DS-ADE population evaluation in Phase I, where each generation involves a full forward-kinematic pass for every candidate in the differential-evolution pool. Despite the higher absolute runtime, two considerations place this overhead in perspective. First, the 61.11 s budget remains approximately 2.6× faster than the particle-filter-based EKF-PF, and stays within a practically acceptable one-minute-scale window for offline recalibration in manufacturing cells. Second, the accuracy return on this time investment is substantial: relative to KC-JVP, the test RMSE drops by 23.46% (from 0.81 mm to 0.62 mm), representing a favorable cost–accuracy tradeoff for applications where sub-millimetre precision is the primary objective. In scenarios demanding shorter cycle times, the DS-ADE population size can be reduced to trade a marginal accuracy loss for proportionally faster execution. From a deployment standpoint, the online controller does not retain the DE population, external archive, or calibration dataset. Runtime memory is limited to the frozen D-H parameters, the compact one-layer GKAN spline coefficients, and transient Jacobian/LM matrices, which makes the online footprint substantially smaller than the offline training state.

#### 3.5.3. Symbolic Parameter Identification

Table 8 lists the kinematic parameters identified by the Evo-NPH framework for the ABB IRB 120. All deviations from the nominal design values (Table 1) remain within physically plausible ranges: link-length and offset corrections fall within ±1 mm, while angular perturbations stay below ±3°, consistent with typical manufacturing and assembly tolerances for this class of manipulator. The calibrated joint offsets $\theta_{0,i}^{\mathrm{cal}}$ absorb both the nominal D-H convention offsets and the residual encoder mounting errors, providing a single consolidated correction per axis.

A distinguishing feature of the proposed framework is the separation between geometric and non-geometric error channels. In conventional least-squares identification, the D-H parameters are the sole free variables and therefore tend to compensate effects they were never designed to capture, such as joint compliance or thermal drift. The Evo-NPH architecture avoids this conflation: the GKAN branch in the NPHJ stage absorbs configuration-dependent non-geometric residuals, so the symbolic parameters in Table 8 encode only the static rigid-body geometry of the kinematic chain. This decoupling preserves the physical interpretability of the identified model and avoids parameter distortion that could degrade accuracy under operating conditions different from those used during calibration.

#### 3.5.4. Ablation Study

Three ablated variants of the Evo-NPH framework were constructed to isolate the contribution of each core component: the joint optimization strategy, the neural compensator architecture, and the evolutionary search mechanism.

- V1 (Sequential Optimization) identifies the geometric D-H parameters via the standard Levenberg–Marquardt algorithm in a first stage, then trains the neural compensator on the frozen residuals. This variant quantifies the gain of simultaneous parameter–network co-optimization over the conventional two-stage pipeline.
- V2 (MLP Substitution) replaces the Graph Kolmogorov–Arnold Network with a Multi-Layer Perceptron of matched parameter count and ReLU activations, while retaining all other components unchanged. The comparison isolates the effect of learnable spline activations versus piecewise-linear units.
- V3 (Single-Strategy DE) adopts a standard Differential Evolution algorithm with a fixed rand-to-pbest/1mutation operator, removing the adaptive dual-strategy selection of DS-ADE. This variant measures the benefit of strategy-level adaptation for escaping local optima.
- V4 (Evo-NPH) is the complete framework combining DS-ADE initialization, the GKAN compensator, and the NPHJ refinement stage.

Table 9 summarizes the results. The most significant accuracy gap appears between the sequential pipeline (V1) and the full framework (V4): decoupling parameter identification from compensator training raises the test RMSE from 0.62 mm to 0.92 mm, a degradation of 32.6%. In the sequential setting, the LM stage has no mechanism to account for non-geometric effects; the resulting geometric parameters therefore partially absorb compliance- and thermal-related errors, which distorts the residual landscape presented to the neural stage. Joint optimization sidesteps this issue by allowing the solver to allocate error correction across symbolic parameters and network weights simultaneously.

Replacing the GKAN with an equivalently sized MLP (V2) increases the test RMSE to 0.84 mm, corresponding to a 26.2% reduction in accuracy relative to V4. The spline-based activations in the Kolmogorov–Arnold formulation offer a richer function space for approximating the smooth, low-frequency structure of kinematic residuals than the piecewise-linear mappings of ReLU networks, which accounts for the observed difference.

Removing the dual-strategy adaptation from the evolutionary engine (V3) yields a test RMSE of 0.79 mm, 21.5% above the full framework. With a single fixed mutation operator, the search lacks the mechanism to shift between explorative and exploitative phases as the population matures; the DS-ADE scheduler addresses this by reallocating strategy probabilities on the basis of recent success history, enabling more reliable convergence toward the global optimum.

Taken together, these results indicate that each of the three components—joint optimization, GKAN architecture, and adaptive differential evolution—provides a distinct and complementary contribution. The joint optimization strategy exerts the largest individual effect, followed by the neural architecture choice and the evolutionary search mechanism.

#### 3.5.5. Comparison with Advanced Differential Evolution Variants

To directly benchmark the proposed DS-ADE optimizer against representative advanced adaptive DE baselines, an additional comparison was conducted using JADE [25], SHADE [26], and L-SHADE [27]. For fairness, all methods optimized the same unified decision vector, used the same initialization bounds and stopping criterion, and were allocated the same total fitness-evaluation budget. The reported metrics include the final objective value, test-set RMSE, convergence generation, wall-clock time, and the standard deviation across 30 independent runs with different random seeds.

The results in Table 10 distinguish the specific contribution of the proposed dual-strategy scheduler from the broader family of adaptive DE algorithms. Among the external baselines, L-SHADE is the strongest competitor, reaching a test RMSE of 0.66 mm. Nevertheless, DS-ADE still achieves the best final objective value (0.337), the lowest test RMSE (0.62 mm), the fewest convergence generations (86), and the smallest run-to-run deviation (0.015). These results indicate that the proposed dual-strategy adaptation remains effective even when compared against modern success-history-based DE variants.

#### 3.5.6. Hybrid Jacobian Convergence Analysis

To isolate the contribution of the Jacobian design itself, an additional inverse-kinematics benchmark was conducted comparing three solvers: analytical Jacobian only ($J_{phy}$), neural Jacobian only ($J_{nn}$), and the full Hybrid Jacobian ($J_{hybrid}$). Each solver was evaluated on the same target set using a common initialization strategy and the same damping factor λ. The reported metrics include mean iteration count, success rate under a 1.0 mm final-residual tolerance, final Cartesian residual, and average per-query solve time.

The results in Table 11 clarify the convergence mechanism of the proposed NPHJ solver. The analytical Jacobian alone preserves rigid-body structure and therefore remains relatively stable, but its final residual is limited to 0.95 mm. In contrast, the neural Jacobian alone lacks the hard kinematic backbone required for globally stable descent, resulting in the lowest success rate (74%) and the largest iteration count (13.2). By combining both terms, the hybrid formulation achieves the best overall convergence profile, with a 98% success rate, only 5.7 average iterations, and a final residual of 0.58 mm, while preserving millisecond-level query time.

#### 3.5.7. Regularization Sensitivity Discussion

A compact sensitivity discussion of the regularization coefficients is summarized in Table 12. The table reports representative coefficient regimes and their practical effect on the balance between geometric plausibility and neural flexibility.

As indicated by Table 12, the selected pair $\left(\lambda_{DH},\lambda_{Res}\right)=\left(10^{-4},5\times 10^{-2}\right)$ lies in a practically balanced regime: smaller $\lambda_{DH}$ values make the symbolic calibration less physically constrained, whereas larger $\lambda_{Res}$ values suppress the neural branch and reduce the framework’s ability to absorb configuration-dependent residuals.

#### 3.5.8. Statistical Significance Analysis

Wilcoxon signed-rank tests were performed to determine whether the accuracy differences between Evo-NPH (M8) and each baseline are statistically meaningful. This non-parametric test compares paired error samples without assuming a particular distributional form, making it appropriate for distance-residual error data whose distribution is generally unknown. For every pair, $R^{+}$ accumulates the ranks of samples where M8 produces a smaller error, while $R^{-}$ accumulates the ranks where the baseline is more accurate; the null hypothesis posits no systematic difference between the two methods.

Table 13 reports the results on both the training set (500 samples, total rank sum $R^{+}+R^{-}=125,250$) and the test set (100 samples, total rank sum 5050). In every comparison, the *p*-value falls below $10^{-3}$, well under the α=0.05 threshold, so the null hypothesis is rejected in all cases. On the test set, $R^{+}$ exceeds $R^{-}$ by at least a factor of four even for the closest competitor KC-JVP (M7), where $R^{+}=4141$ versus $R^{-}=909$. The ratio widens steadily as the baseline accuracy decreases: for LM-PF (M4), the most distant baseline, $R^{+}$ reaches 4734 against $R^{-}=316$. A consistent pattern holds on the training set. These results confirm that the observed improvements are not attributable to sampling variability and that the Evo-NPH framework provides a statistically significant advantage over all evaluated baselines across both data partitions.

## 4. Conclusions

This study addresses the competing requirements of global convergence and computational tractability in industrial robot calibration by introducing the Evo-NPH framework. The architecture rests on three interconnected components: a joint optimization formulation in which geometric D-H parameters and neural compensator weights are evolved concurrently under a unified objective—mirroring the biological principle of brain–body co-adaptation—thereby circumventing the error accumulation that arises when identification and compensation are performed sequentially; a Graph Kolmogorov–Arnold Network whose learnable spline activations approximate the smooth, low-frequency structure of non-geometric residuals more faithfully than conventional piecewise-linear perceptrons; and a Neuro-Physical Hybrid Jacobian solver that exploits the closed-form gradient of the learned compensator to deliver deterministic, millisecond-level correction updates suitable for closed-loop control.

The framework was validated experimentally on an ABB IRB 120 six-axis manipulator using 600 spatially stratified measurement configurations (500 training, 100 test). Across all evaluation metrics the Evo-NPH method surpasses seven published baselines spanning classical geometric identification, recursive Bayesian estimation, metaheuristic search, and data-driven compensation. On the test partition, the proposed method records an RMSE of 0.62 mm, an STD of 0.59 mm, and a maximum error of 0.83 mm, corresponding to an 86.75% reduction relative to the uncalibrated baseline and a 47.01% reduction relative to the geometric-only SGC-LM calibration. Against the strongest competitor, KC-JVP, the error is further lowered by 23.46%.

Ablation experiments isolate the contribution of each architectural choice. Replacing the GKAN with a parameter-matched MLP raises the test RMSE by 26.2%, while reverting from joint optimization to a conventional sequential pipeline degrades accuracy from 0.62 mm to 0.92 mm. Wilcoxon signed-rank tests yield p<0.001 for every pairwise comparison, confirming that the observed improvements are not attributable to sampling variability.

Several open questions merit further study. The current validation is limited to an open-chain serial manipulator; extending the framework to closed-chain parallel kinematic structures would broaden its industrial applicability. The offline DS-ADE initialization could be reformulated as an incremental learning procedure in which the GKAN spline coefficients are periodically updated to track long-term mechanical degradation. Hardware acceleration of the Hybrid Jacobian evaluation, for instance through FPGA implementation, would further reduce the online compensation latency and support high-bandwidth servo loops in next-generation manufacturing systems. For parallel robots in particular, the present serial-chain formulation would need to be extended to account for passive joints, loop-closure constraints, and multiple coupled constraint Jacobians. Likewise, although the current experiments rely on a draw-wire encoder and an ABB IRB 120 platform, the framework itself depends only on a differentiable forward model and an observation operator; in principle, alternative sensing modalities such as laser trackers, vision systems, or ball-bar measurements can be accommodated by redefining the measurement model in the objective function. Cross-platform validation on additional robot architectures and sensors remains an important next step.

## Figures and Tables

**Table 1:** Nominal D-H kinematic parameters of the ABB IRB 120 robot.

Joint *i*	$\alpha_{i}$ (deg)	$a_{i}$ (mm)	$d_{i}$ (mm)	$\theta_{i}$ (deg)
1	−90	0	290	0
2	0	270	0	−90
3	−90	70	0	0
4	90	0	302	0
5	−90	0	0	0
6	0	0	72	0

**Table 2:** Hyperparameter configurations for comparative methods and the proposed framework.

Method	Key Hyperparameters	Value
M1	Optimization Solver	Levenberg–Marquardt (LM)
M2	Structure (Hidden neurons)	40
(ES-RBFNN)	σ/Learning rate η	ES-derived/0.01
M3	Particle count $N_{p}$	500
(EKF-PF)	Noise cov. Q/*R*	$10^{-5}I$/$10^{-2}$
M4	LM iterations/PF particles	50/200
(LM-PF)	Initialization	LM-seeded
M5	Swarm sizes (QPSO-1/2)	40/40
(DQPSO)	ANFIS MFs/Max iter.	3 Gauss/100
M6	Pop. size/Switch prob. *p*	30/0.8
(BFPA)	Levy exponent α	1.5
M7	RPSO swarm/Inertia *w*	50/0.9→0.4
(KC-JVP)	DCFNN structure	[6→128→64→6]
	**Phase I (DS-ADE)**	
	Pop. size/Max gen.	48/300
M8	Memory *H*/*p*-best	4/0.12
(Ours, Evo-NPH)	GKAN Grid *G*/Layers	5/1
	**Phase II (NPHJ)**	
	λ/α	$10^{-2}$/1.0

**Table 3:** Workflow of the DS-ADE global optimization and NPHJ control framework.

Procedure	Complexity *
**Input:** Calibration dataset $D={\left\{\left(q_{k},z_{k}^{meas}\right)\right\}}_{k=1}^{N}$; Search bounds; Target $p_{target}$.	—
**Phase I: Offline Global Joint Optimization (DS-ADE)**	
1: **Initialize** Population $P_{0}$ with NP individuals $\Theta_{i}$	O(NP·D)
2: **for** g=1 to $G_{max}$ **do**	$\times G_{max}$
3: // 1. Dual-Strategy Mutation	—
4: Select Strategy $S_{k}$ based on prob $p_{1}$ (Equation (14))	O(1)
5: Generate Mutant $v_{i,g}$ via Equation (12) or Equation (13)	O(NP·D)
6: // 2. Crossover & Evaluation	—
7: Generate Trial $u_{i,g}$ (Binomial Crossover)	O(NP·D)
8: Evaluate Fitness $J(u_{i,g})$ on Dataset D (Equation (9))	$O(NP\cdot N\cdot C_{FK})$
9: Selection: $\Theta_{i,g+1}\leftarrow u_{i,g}$ if improved	O(NP)
10: // 3. Parameter Adaptation	—
11: Update Memory M, sample $\left(F_{i},CR_{i}\right)$, and update $p_{1}$	O(H)
12: **end for**	—
13: **Output:** Optimal Hybrid Parameters $\Theta^{*}={\left[\Psi^{*⊤},W_{GKAN}^{*⊤}\right]}^{⊤}$	—
**Phase II: Online Real-Time Control (NPHJ Solver)**	
14: **Initialize** $q\leftarrow q_{init}$, damping λ	O(n)
15: **while** ∥Δq∥>ϵ **do**	$\times T_{iter}$
16: // 1. Hybrid Forward Evaluation (Frozen $\Theta^{*}$)	—
17: $p_{est}=K_{rigid}\left(q\right)+N_{GKAN}\left(q\right)$	$O(C_{FK})$
18: Tracking Error: $r=p_{target}-p_{est}$	O(1)
19: // 2. Gradient Fusion (Hybrid Jacobian)	—
20: $J_{phy}=\mathrm{CrossProd}\left(z,p\right)$ (Analytical)	O(n)
21: $J_{nn}=\mathrm{Autograd}\left(N_{GKAN}\right)$ (Neural)	$O(C_{FK})$
22: $J_{hybrid}=J_{phy}+J_{nn}$ (Hybrid Jacobian)	O(n)
23: // 3. Deterministic Update (LM)	—
24: Solve $\left(J_{hybrid}^{⊤}J_{hybrid}+\lambda I\right)\Delta q=J_{hybrid}^{⊤}r$	$O(n^{3})$
25: Update q←q+αΔq	O(n)
26: **end while**	—
**Final Output:** Compensated Joint Command $q_{cmd}$	—

* *D*: Param dim; *N*: Data size; NP: Pop size; *H*: memory size; $C_{FK}$: Hybrid fwd cost (∝L·G).

**Table 4:** Technical specifications of the displacement measurement system.

Parameter	Value
Output Signal Type	Digital Pulse
Input Voltage	DC 5–24 V
Measurement Stroke	2000 mm
Max. Retraction Velocity	1000 mm/s
Cable Tension Force	5 N
Linearity Error	0.05% Full Scale
Resolution	0.004 mm
Operating Temperature	−25 °C∼+85 °C

**Table 6:** Quantitative performance comparison of calibration algorithms on training and testing datasets.

Method	Training Set (500 Samples)			Test Set (100 Samples)		
	RMSE	STD	MAX	RMSE	STD	MAX
Before Calibration	4.68	2.20	8.88	4.68	2.24	8.55
M1 (SGC-LM) [21]	0.61	0.51	1.18	1.17	1.16	1.67
M2 (ES-RBFNN) [14]	1.36	1.36	1.73	1.38	1.28	1.48
M3 (EKF-PF) [29]	1.27	1.25	2.33	1.26	1.21	1.55
M4 (LM-PF) [30]	0.76	0.66	1.53	1.54	1.53	1.89
M5 (EKF-DQBPSO) [31]	0.77	0.59	1.31	1.06	1.01	1.36
M6 (ANN-BFPA) [32]	0.54	0.46	1.14	0.99	0.97	1.07
M7 (KC-JVP) [28]	0.62	0.53	1.03	0.81	0.73	1.06
M8 (Evo-NPH, Ours)	**0.31**	**0.24**	**0.87**	**0.62**	**0.59**	**0.83**

Bold values indicate the best performance in each column.

**Table 7:** Computational cost of all calibration methods (evaluated on 600 measurement samples).

Method	Iterations	Time (s)
M1 (SGC-LM) [21]	13	30.97
M2 (ES-RBFNN) [14]	11	36.56
M3 (EKF-PF) [29]	31	160.96
M4 (LM-PF) [30]	62	45.84
M5 (EKF-DQBPSO) [31]	20	21.23
M6 (ANN-BFPA) [32]	236	23.01
M7 (KC-JVP) [28]	199	27.50
M8 (Evo-NPH, Ours)	86	61.11

**Table 8:** Calibrated D-H parameters of the ABB IRB 120 identified by the proposed Evo-NPH framework.

Joint *i*	$a_{i}$ (mm)	$d_{i}$ (mm)	$\alpha_{i}$ (°)	$\theta_{0,i}^{\mathrm{cal}}$ (°)
1	−0.21	289.38	−89.59	0.75
2	270.05	0.39	0.11	−89.88
3	69.27	0.98	−90.27	−0.16
4	−0.65	301.82	91.02	−0.84
5	−0.67	−0.07	−89.83	0.64
6	0.81	71.49	0.27	0.68

**Table 9:** Ablation results showing the contribution of each Evo-NPH component.

Variant	Training Set			Test Set		
	RMSE	STD	MAX	RMSE	STD	MAX
V1: Sequential Opt.	0.55	0.48	1.12	0.92	0.89	1.25
V2: MLP Substitution	0.42	0.39	0.95	0.84	0.81	1.16
V3: Single-Strategy DE	0.45	0.41	1.05	0.79	0.75	1.10
V4: Proposed (Evo-NPH)	**0.31**	**0.24**	**0.87**	**0.62**	**0.59**	**0.83**

All values in mm. Bold values indicate the best performance in each column.

**Table 10:** Comparison of DS-ADE with advanced adaptive DE variants on the joint calibration problem.

Method	Final Obj.	Test RMSE	Conv. Gen.	Time (s)	Std.
JADE [25]	0.405	0.73	124	65.2	0.033
SHADE [26]	0.382	0.69	107	62.1	0.026
L-SHADE [27]	0.358	0.66	95	58.4	0.021
DS-ADE (Ours)	**0.337**	**0.62**	**86**	61.11	**0.015**

All methods were evaluated under the same unified-vector formulation and stopping budget. Bold values indicate the best performance in each column.

**Table 11:** Convergence comparison of analytical, neural, and Hybrid Jacobian solvers.

Solver	Success Rate	Avg. Iter.	Final Residual	Time/Query
Analytical only ($J_{phy}$)	89%	8.6	0.95	0.81
Neural only ($J_{nn}$)	74%	13.2	1.21	1.09
Hybrid ($J_{hybrid}$)	**98%**	**5.7**	**0.58**	1.15

All solvers were evaluated using the same target set, initialization rule, and a 1.0 mm stopping tolerance. Bold values indicate the best performance in each column.

**Table 12:** Representative sensitivity regimes of the regularization coefficients in the unified objective.

Case	$\lambda_{\mathrm{DH}}$	$\lambda_{\mathrm{Res}}$	Primary Effect
Weak geometric prior	$10^{-5}$	$5\times 10^{-2}$	Greater symbolic freedom; weaker physical constraint.
Balanced setting (used)	$10^{-4}$	$5\times 10^{-2}$	Best trade-off between physical plausibility and residual flexibility.
Strong geometric prior	$10^{-3}$	$5\times 10^{-2}$	More symbolic rigidity; the residual branch must absorb larger mismatch.
Strong residual suppression	$10^{-4}$	$10^{-1}$	Stronger residual penalty; reduced neural correction capacity.

Selected operating point: $\left(10^{-4},5\times 10^{-2}\right)$.

**Table 13:** Wilcoxon signed-rank test results comparing Evo-NPH (M8) against each baseline on the training set (500 samples) and the test set (100 samples).

Comparison	Training Set			Test Set		
	$R^{+}$	$R^{-}$	p-Value	$R^{+}$	$R^{-}$	p-Value
M8 vs. M1 (SGC-LM)	112,169	13,081	<0.001	4473	577	<0.001
M8 vs. M2 (ES-RBFNN)	124,207	1043	<0.001	4565	485	<0.001
M8 vs. M3 (EKF-PF)	124,659	591	<0.001	4521	529	<0.001
M8 vs. M4 (LM-PF)	115,273	9977	<0.001	4734	316	<0.001
M8 vs. M5 (EKF-DQBPSO)	115,093	10,157	<0.001	4395	655	<0.001
M8 vs. M6 (ANN-BFPA)	111,252	13,998	<0.001	4299	751	<0.001
M8 vs. M7 (KC-JVP)	111,963	13,287	<0.001	4141	909	<0.001

Significance level α=0.05. All *p*-values computed via the exact Wilcoxon distribution.

## Data Availability

The data presented in this study are available on request from the corresponding author. The data are not publicly available due to institutional policy restrictions.
